# Does sports industry development improve regional public health? A cross-regional heterogeneous study in China

**DOI:** 10.3389/fpubh.2026.1770636

**Published:** 2026-02-13

**Authors:** Shuxiong Song

**Affiliations:** Department of Physical Education, Anhui Science and Technology University, Chuzhou, Anhui, China

**Keywords:** China, panel data, public health, regional heterogeneity, sports industry development, threshold effect

## Abstract

This study investigates whether sports industry development improves regional public health in China, with particular attention to potential nonlinear threshold effects and regional heterogeneity. Using panel data from 30 Chinese provinces over the period 2010–2024, Hansen’s panel threshold regression model is employed to examine the relationship between sports industry development (measured as percentage of GDP) and life expectancy, supplemented by instrumental variable estimation and system GMM approaches to address endogeneity concerns. The results reveal a significant single threshold effect at 0.96% of GDP (95% CI: 0.89–1.04), with the promotion coefficient increasing from 0.312 to 0.369 (+18.3%) after crossing this threshold. Regional analysis uncovers substantial heterogeneity: Eastern China exhibits a linear relationship without threshold effects, while Central and Western regions demonstrate distinct thresholds at 1.06 and 0.79%, respectively, with effect enhancements of 13.3 and 26.4% after crossing these thresholds. These findings suggest that sports industry development significantly promotes public health through nonlinear mechanisms that vary across regional development stages. Policy implications indicate that differentiated strategies should prioritize sports industry investment in underdeveloped Western regions where marginal health benefits are greatest.

## 1 Introduction

The promotion of population health is considered a pivotal governmental agenda presently around the world, particularly due to an increasing recognition of the importance of economic development channels in influencing healthier outcomes. The World Health Organization reports that physical inactivity increases the risk of premature mortality by 20–30% and contributes significantly to the global burden of noncommunicable diseases (1). Governments worldwide have increasingly recognized the sports industry as a potential pathway for improving population health. Yet, while the health benefits of individual physical activity participation are well established, the macro-level relationship between sports industry development and regional public health outcomes remains underexplored. Regarding the Chinese context, the tremendous growth observed in the sport market during the previous 10 years is accompanied by improvements in population health, raising critical questions about the underlying mechanisms. It has been found in the prior literature that the contribution to the GDP made by the sporting industry grew from around 0.55% in 2010 to over 1.15% in 2023 (2). This growth pattern corresponds to overall strategic plans that give priority to the Healthy China 2030 plan, which specifically relates the promotion of the sporting industry to the aim of improving the health status of the populace.

Despite the growing awareness of this issue among policymakers, the exact relationship between sports industry growth and public health is rather unclear. This study addresses a specific question: Does sports industry development at the regional level improve public health, and does this relationship exhibit nonlinear threshold effects that vary across different development stages? Even straightforward logic suggests that the higher the development of the sports sector, the healthier the population because of increased exercise and sports infrastructure (3). In addition, existing research has not extensively explored the non-linear character of the relationship between public health expenditures and the potential differentials of health outcomes at different development stages (4). Some existing research on the expenditures has identified the existence of a threshold effect between additional expenditures and different development stages (5). Such patterns have been identified in studies of public health and economic development in Chinese cities (6). Some current evaluations show great regional disparities in the implementation of China’s national fitness policy and its health impacts, while the Healthy China 2030 policy has demonstrated heterogeneous effects across geographic areas (7).

**Figure 1:**
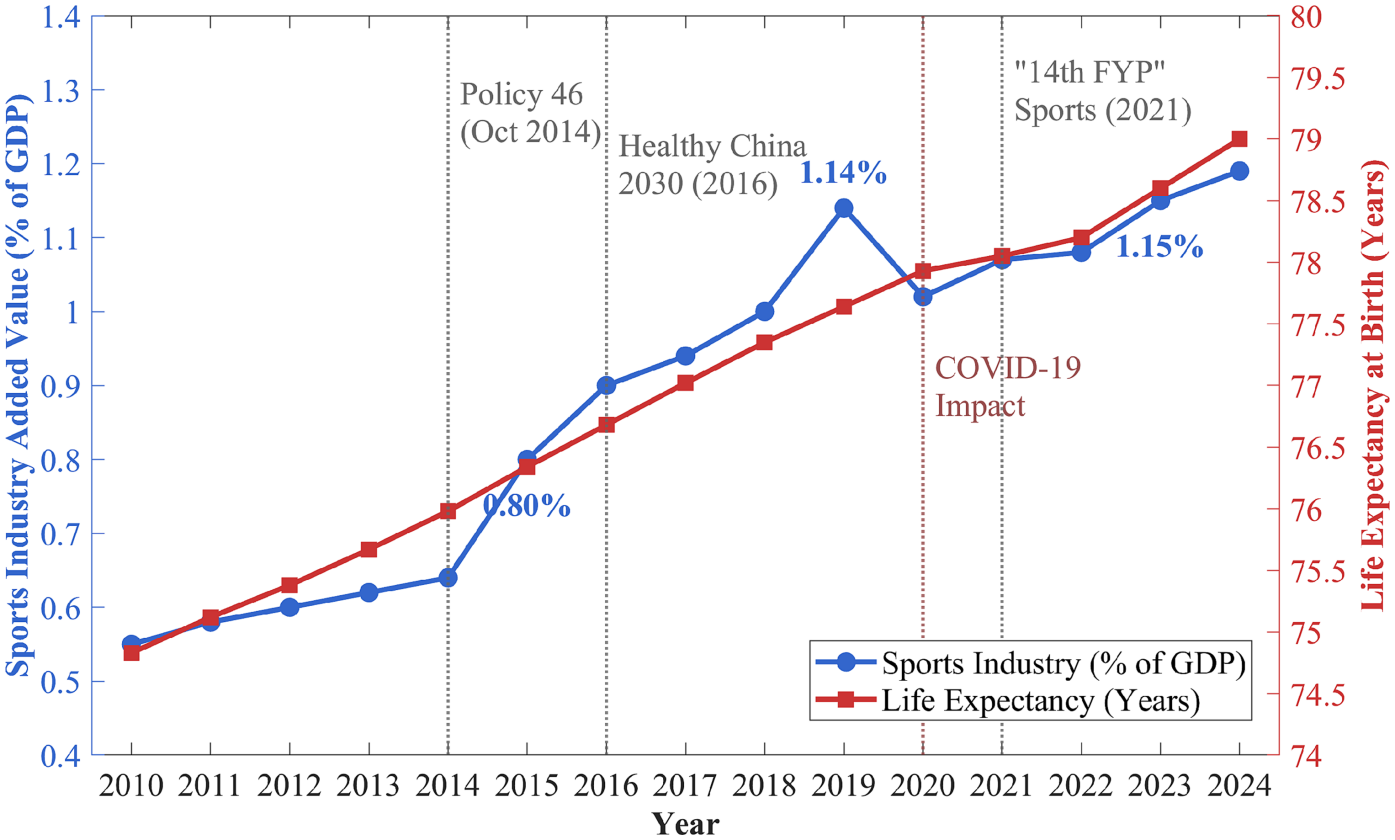
Trends in China’s sports industry development and public health (2010–2024).

The study systematically examines the connection between sports industry development and health outcomes, focusing on Chinese provinces. In particular, it carefully explores possible thresholds in this connection. This research contributes to existing literature in three ways. It applies Hansen’s panel threshold regression to identify nonlinear patterns that linear models would overlook. It conducts comparative analysis across Eastern, Central, and Western China to examine how regional development contexts shape health returns to sports investment. It also employs instrumental variables and system GMM estimation to address endogeneity concerns and strengthen causal inference. As illustrated in Figure 1, both sports industry growth and life expectancy showed a steadily increasing trend over the period that served as an empirical foundation for conducting an analysis.

As shown in Figure 1, the GDP from the sports industry has grown from 0.55% in 2010 to 1.19% in 2024, taking just 15 years to more than double in size, following the direction indicated by the State Council Policy Document No. 46 in 2014, where the sports industry was set to achieve staggering goals that involved integrating with the health industry. Life expectancy rose from 74.83 years in 2010 to 79.00 years in 2024, a boost of more than 4 years. The COVID-19 outbreak in 2020 momentarily influenced both, as the percentage of the sports industry out of the total industry dropped from 1.14 to 1.02%, while the rate of increase in life expectancy reduced. However, the signs of recovery emerged in 2021–2022, corresponding with the 14th Five-Year Plan for Sports, during which the indicators returned to the trend observed prior to the pandemic. While aggregate data shown here suggest a positive relationship, a causal relationship requires careful econometric analysis of confounding variables and reverse causality, which is the approach taken in this study.

## 2 Literature review

### 2.1 Sports industry development and public health

Research examining the relationship between sports industry development and public health has expanded considerably in recent years. Studies on coupling coordination between the sports industry and health service industry showed a positive interaction, which suggests that these two industries are complementary to each other, impacting wellbeing (8). The level of coupling coordination shows great variation between Chinese provinces, with more advanced provinces usually having a greater level of coupling coordination. This finding suggests that the health advantages that can be gained with development in sports industry may have a foundation in simultaneous investments in health service infrastructure. At the macro level, Luo and Chen (9) employed a spatial Durbin model to demonstrate that regional sports industry agglomeration exerts positive spatial spillover effects on residents’ health in China. International evidence also supports this relationship. Zhai et al. (4) found that sports industry development significantly improves quality of life and promotes regional development across multiple countries. These macro-level findings complement research on the mechanisms linking sports development to health outcomes.

The integration of sports and health sectors has been identified as a driver of industrial upgrading, generating both direct and indirect health benefits (10). This perspective argues that the sports sector, in a similar manner as other Chinese sectors, provides positive externalities that multiply health effects through overall improved standards of life and services. Regional research evaluating the fitness and health development of nations in Eastern China has revealed considerable variation among provinces regarding levels of coordination, emphasizing the importance of context (11). Even within the relatively developed eastern region, significant disparities exist in the effectiveness of sports-health integration. These regional disparities within China mirror broader international patterns.

From a global perspective, the role of sporting activities in contributing to sustainable development goals has increasingly been emphasized, especially those related to health. Narrative reviews related to the localization of health-related SDGs sporting activities in China have identified the different ways in which sporting activities contribute to the wellbeing of the people (12). There are systematic reviews that have synthesized the evidence regarding the role of sport in the SDGs and public health, highlighting positive trends while showing considerable levels of heterogeneity (13). The use of big data analytics in studies has made it possible to investigate the role of the sports economy in a more comprehensive manner in relation to the health industry (14). However, the majority of existing studies rely on correlation or linear regression methods, potentially overlooking threshold effects and nonlinear relationships between sports industry development and health outcomes. This methodological limitation motivates an examination of nonlinear dynamics, which has received greater attention in the broader literature on economic growth and health.

### 2.2 Economic growth and health: nonlinear relationships

A substantial body of research has examined nonlinear relationships between economic development and health indicators, consistently finding that the health returns to economic growth vary across different stages of development (15, 16). These studies indicate that linear models may underestimate or overestimate the true effects depending on contextual conditions. In the Chinese context, some critical thresholds have been identified for the effects of economic growth and public health by cross-regional research, and the effect of economic growth on health varies accordingly depending on different levels of economic development (17). The above results show that marginal health returns to economic development are conditional on baselines with different mechanisms above and below certain thresholds. Panel threshold analysis on relationships between health and economic growth has established that health returns differ above various income thresholds with some showing signs of diminishing returns at higher stages of development, and health returns in other areas are magnified (18).

Research on human capital structure reveals nonlinear feedback mechanisms in public health provision, with the effect of advanced human capital on health improvements becoming more pronounced once educational attainment crosses certain thresholds (15). Such findings show that the health outcomes of development efforts could be driven by the conditions met by human capital. Government intervention emerges as an important moderating influence on the relationship that exists between economic circumstances and health outcomes. Threshold analyses suggest that the health-protecting role of government intervention is influenced by either the severity or the timing of the intervention (19). This has important implications for the nature of health gains that can be yielded through the development of any industry such as sports. Demographic factors also interact with these threshold dynamics.

Aging population is another area where threshold effects exist. Panel threshold regression results reveal the existence of systematic impacts on the economic effects of demographic transition depending on development characteristics in Chinese regions (20). The methodology employed in aging and health studies presents a conceptual approach in analyzing the existence of threshold effects in the sports and health nexus. These results suggest that threshold methodologies are a necessary tool in understanding the nonlinear relationships between economic conditions and health outcomes. Extending these threshold methodologies to the sports-health nexus represents a logical progression of this research agenda, yet such applications remain scarce.

### 2.3 Regional heterogeneity in health outcomes

Health disparities across different regions have continued to pose a challenge to public health policies in China. Using temporal convergence and decomposition analysis, researchers have assessed trends in provincial health inequality between 1990 and 2019 (21). The factors influencing regional disparity in health status are complex and include variances in economic development, healthcare resources, environmental factors, and healthcare behavior. Studies of necessary medical services and healthcare in the provinces of China highlight regional disparity in healthcare provision in relation to development along the eastern coast as opposed to the inner regions, which could affect the impact of development in the sports industry on health status (21). Beyond the east–west divide, demographic characteristics also shape regional health patterns.

Research on ethnic minority areas has identified distinct patterns in medical resource allocation and healthcare accessibility that require region-specific interventions (22). These patterns suggest that the relationship between development outlays and health outcomes tends to differ across areas that have divergent demographic characteristics. Technological and efficiency heterogeneity is emerging as a factor that affects the performance differences in the healthcare system across various regions. Studies on the total factor productivity changes in the healthcare system in China indicate obvious heterogeneity in technological progress (23). These efficiency differentials have implications for how regional development initiatives translate into health outcomes.

Spatiotemporal studies of health-related poverty show that health poverty persists in some regions due to socioeconomic factors, suggesting the need for targeted policies in underdeveloped areas (24). In the sports sector, empirical analysis of the spatial heterogeneity of National Fitness Plans shows the implementation effectiveness of the eastern provinces is generally higher than that of the central and western provinces. This implies a similar pattern may also exist regarding the health effect of sports industry development (25). These findings suggest that the health effects of sports industry development are likely to vary substantially across regions, yet existing research has not systematically examined this heterogeneity using appropriate econometric methods.

### 2.4 Research gaps and contributions

Despite considerable advances in understanding the determinants of population health and the economic dynamics of sports development, several critical gaps remain in the literature. Although the beneficial relationship between sports participation and health has been well established at the individual level, the role of sports industry development at the aggregate regional level has received limited research attention. The potential existence of threshold effects within the sports-health relationship has not been examined using econometric tools capable of identifying nonlinear patterns. Regional heterogeneity across China’s diverse economic systems remains largely unexplored, and existing studies have generally not addressed endogeneity concerns arising from reverse causality or omitted variables. These limitations constrain the policy relevance of existing findings.

Previous studies have documented divergent patterns of economic growth and health expenditure across countries, emphasizing the complexity of these relationships and the need for context-specific analysis (26). While panel causality tests have established correlations between health spending and economic growth, the specific contribution of sports industry investment has not been isolated from broader health expenditure effects (27). This study addresses these gaps by employing Hansen’s panel threshold regression, combined with instrumental variable and system GMM estimation, to analyze the sports-health relationship across 30 Chinese provinces from 2010 to 2024. This approach enables the identification of threshold values at both national and regional levels while addressing endogeneity concerns, thereby providing credible estimates of how health returns vary across different stages of sports industry development.

## 3 Theoretical framework and hypotheses

This study draws on Grossman's (28) health production function, which conceptualizes health as a durable capital stock that individuals produce through various inputs including time and market goods. Within this framework, sports industry development represents a supply-side factor that expands the availability of health-producing inputs at the regional level. Building on this theoretical foundation, three mechanisms link sports industry development to population health outcomes. Sports participation serves as a direct health investment that reduces chronic disease risk (46). Sports facility accessibility lowers the transaction costs of health production by reducing travel time and financial barriers to physical activity (29). Health consciousness, shaped by exposure to sports culture and marketing, influences individuals’ preferences for health-enhancing behaviors (9). Empirical evidence supports these mechanisms. Systematic reviews confirm that physical activity reduces cardiovascular, metabolic, and cognitive disease risks (30, 31). Population-level studies demonstrate significant associations between physical activity and chronic disease prevention (32). In China, sports infrastructure development has been identified as a policy priority for achieving national health goals (33).

**Figure 2:**
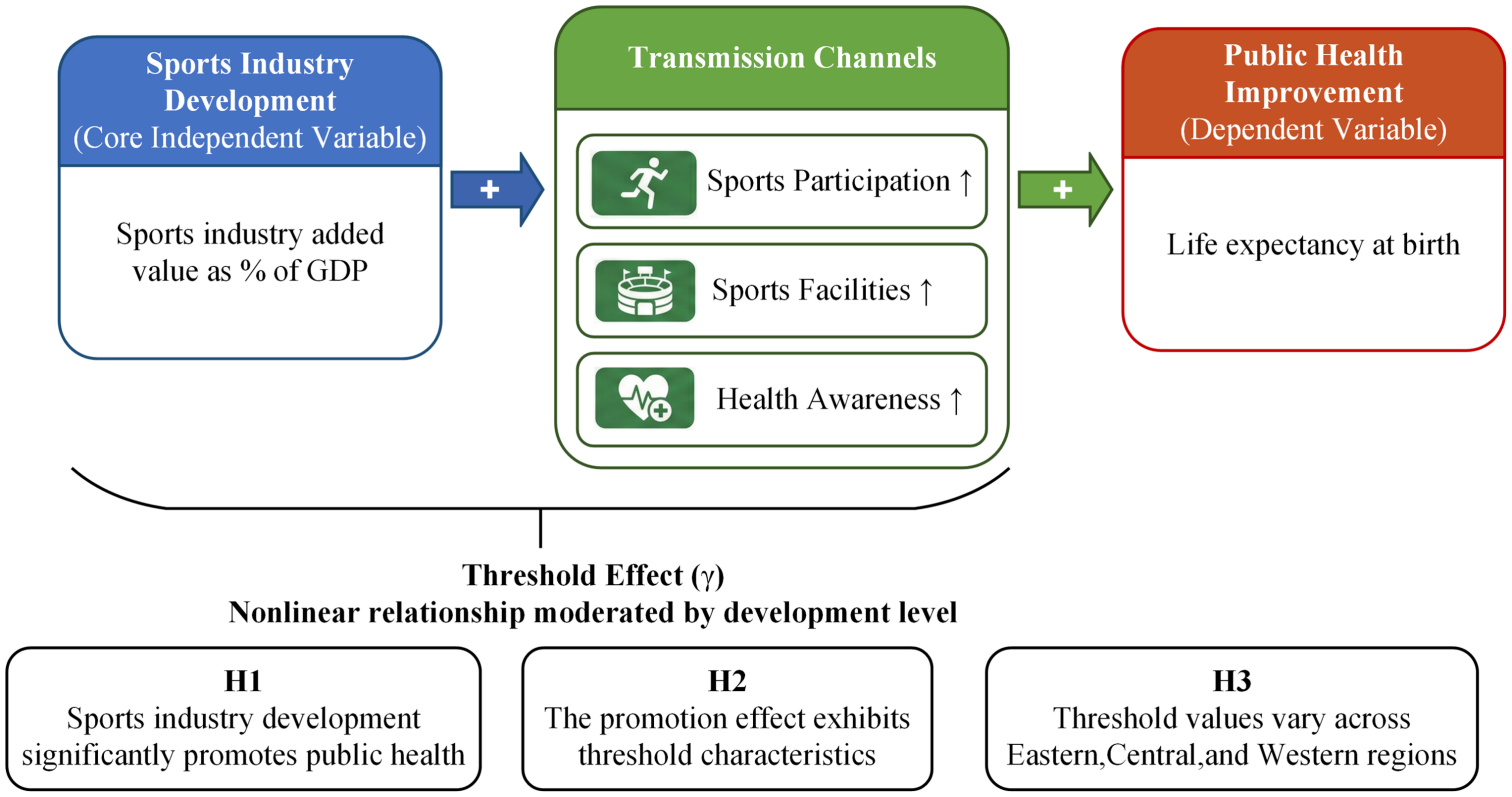
Theoretical framework: sports industry development and public health.

The framework incorporates threshold effects, drawing on the theoretical insight that returns to health investment may exhibit nonlinear patterns across development stages (15). In the early stages of sports industry development, health spillovers remain constrained by insufficient infrastructure density and limited household purchasing power. Once development crosses critical thresholds, health promotion mechanisms become more efficient through network externalities that increase the value of sports participation, economies of scale that reduce per-capita service costs, and cultural accumulation effects that normalize health-conscious behaviors. Recent empirical studies have confirmed such threshold patterns in health-economy relationships across multiple country contexts (16). The theoretical model is illustrated graphically in Figure 2 showing threshold values, whose estimation is required through empirical work.

As shown in Figure 2, sports industry development (expressed as a percentage of GDP) serves as the primary independent variable and affects public health outcomes through three mediating pathways. The threshold effect (*γ*) stands for the level at which enhanced health-supportive outcomes occur. The framework hypothesizes that threshold values vary across regions due to differences in baseline infrastructure, health service capacity, and population health behaviors, with the specific values (*γ*_Western_, *γ*_National_, *γ*_Central_) to be empirically determined rather than assumed *a priori*. Based on this theoretical framework, three hypotheses are proposed.

*H1*: Sports industry development has a significant positive effect on public health outcomes.

*H2*: The relationship between sports industry development and public health exhibits threshold effects, with the health-promoting effect strengthening after development crosses critical levels.

*H3*: Threshold values differ across Eastern, Central, and Western China due to heterogeneous baseline conditions in infrastructure endowment, health service capacity, and population health behaviors.

## 4 Research design

### 4.1 Data sources and sample

The analysis uses panel data covering 30 Chinese provinces, autonomous regions, and municipalities from 2010 to 2024, yielding 450 province-year observations. Tibet is excluded due to data availability constraints. The study period encompasses several major policy milestones in China’s sports sector, including State Council Policy Document No. 46 in 2014, the Healthy China 2030 initiative in 2016, and the 14th Five-Year Plan for Sports Development in 2021. This temporal coverage enables examination of both pre- and post-policy dynamics. Data for 2023 and 2024 are provisional and subject to minor revisions in subsequent official publications.

Life expectancy at birth serves as the dependent variable. Although life expectancy is a slow-moving indicator that accumulates health improvements over time, it remains appropriate for this analysis. The 15-year panel captures substantial variation in Chinese provincial life expectancy, with improvements ranging from 2 to 6 years across provinces during the study period. Life expectancy responds to factors that sports industry development plausibly influences, including chronic disease prevalence and healthcare utilization patterns. The annual panel structure allows detection of gradual shifts in health trajectories associated with sustained sectoral development rather than immediate year-to-year effects.

Sports industry data are obtained from the National Bureau of Statistics and the General Administration of Sport of China, which jointly publish annual statistics on the size and structure of the sports market. Previous research has validated these statistics as reliable data sources for examining government sports expenditure and physical activity participation (34). Public health data, including life expectancy at birth, are extracted from China Health Statistical Yearbooks and National Health Commission publications. Control variables are compiled from China Statistical Yearbooks, Provincial Statistical Yearbooks, and China Environmental Statistical Yearbooks. Sports consumption data are drawn from studies on consumption trends (35).

The regional classification follows the standard three-region framework used by Chinese statistical authorities. Eastern China comprises 11 provinces: Beijing, Tianjin, Hebei, Liaoning, Shanghai, Jiangsu, Zhejiang, Fujian, Shandong, Guangdong, and Hainan. Central China includes eight provinces: Shanxi, Anhui, Jiangxi, Henan, Hubei, Hunan, Jilin, and Heilongjiang. Western China encompasses 11 provinces: Inner Mongolia, Guangxi, Chongqing, Sichuan, Guizhou, Yunnan, Shaanxi, Gansu, Qinghai, Ningxia, and Xinjiang. This classification captures varied patterns in economic development, industrial structure, and health infrastructure that are expected to condition the sports-health relationship.

### 4.2 Variable definitions

The dependent variable is life expectancy at birth (LE), measured in years. This indicator represents the average number of years a newborn would be expected to live if current mortality patterns remained constant throughout life, providing a comprehensive measure of population health status. Life expectancy is widely recognized as an aggregate indicator for public health and has been used extensively in research examining relationships between urbanization and health expenditure outcomes (36). Studies assessing the influence of household health expenditures on subjective wellbeing have also supported the validity of this indicator as an appropriate health outcome measure (37).

The key independent variable and threshold variable is the development level of the sports industry (SI), measured as the percentage of regional GDP contributed by sports industry value added. This variable captures the relative importance of the sports sector to the regional economy, reflecting both supply-side capacity (sports facilities, services, and manufacturing) and demand-side utilization (sports consumption and participation). The ratio specification accounts for differences in overall economic size across provinces, facilitating cross-regional comparisons.

Seven control variables are included following the framework of existing literature. Economic development level (lnPGDP) is measured as the natural logarithm of per capita GDP. Medical expenditure (ME) captures per capita health spending. Education level (EDU) is measured by average years of schooling. Urbanization rate (URB) represents the share of urban population. Environmental quality (PM25) is measured by annual average PM2.5 concentration. Population aging (AGE) is captured by the share of population aged 65 and above. Medical resources (MR) is measured by health technicians per 1,000 population. The definitions and data sources for all variables are presented in Table 1.

**Table 1:** Variable definitions and data sources.

Variable	Definition	Unit	Data source
LE	Life expectancy at birth	Years	National Health Commission
SI	Sports industry added value/GDP	%	National Bureau of Statistics
lnPGDP	Log of per capita GDP	Log yuan	China Statistical Yearbook
ME	Per capita health expenditure	Yuan	China Health Statistical Yearbook
EDU	Average years of schooling	Years	China Statistical Yearbook
URB	Urban population share	%	China Statistical Yearbook
PM25	Annual average PM2.5	μg/m^3^	Environmental Statistical Yearbook
AGE	Population aged 65+	%	China Statistical Yearbook
MR	Health technicians per 1,000	Persons	China Health Statistical Yearbook

### 4.3 Descriptive statistics

Table 2 summarizes descriptive statistics for the full sample. The average life expectancy is 76.58 years, with a standard deviation of 2.79 years, ranging from 68.17 to 84.63 years. This substantial variation provides a sound basis for examining health determinants. The sports industry development averages 0.94% of GDP, ranging from 0.32 to 2.86%. The maximum value (2.86%) corresponds to observations from earlier years in economically advanced provinces. The National Bureau of Statistics refined the sports industry statistical classification in 2019, narrowing the scope of included activities. For comparison, Shanghai’s sports industry value added accounted for 1.86% of GDP in 2024 under the current classification. This measurement change does not affect the validity of within-panel comparisons, as all provinces follow the same classification standards within each year. The coefficient of variation for SI is 0.48, indicating sufficient variation for threshold analysis. The control variables also exhibit substantial variation: per capita GDP ranges from approximately 10,000 to over 180,000 yuan, urbanization rates span from 32% to nearly 90%, and PM2.5 concentrations vary from 8 to 89 μg/m^3^.

**Table 2:** Descriptive statistics (full sample, *N* = 450).

Variable	Mean	Std. Dev.	Min	Max	Obs
LE	76.58	2.79	68.17	84.63	450
SI	0.94	0.45	0.32	2.86	450
lnPGDP	10.68	0.58	9.21	12.15	450
ME	4256.32	2187.45	856.24	12458.67	450
EDU	9.12	1.08	6.45	12.86	450
URB	58.67	13.24	32.45	89.60	450
PM25	38.56	16.78	8.24	89.45	450
AGE	11.86	3.12	5.67	21.34	450
MR	6.45	2.34	2.86	14.56	450

Regional comparisons reveal systematic differences across the three macro-regions, as shown in Table 3. The average life expectancy in Eastern provinces is 78.65 years, followed by Central regions at 76.92 years and Western regions at 74.18 years. This ordering largely reflects overall economic development levels. Sports industry development shows similar patterns, averaging 1.31% of GDP in Eastern provinces compared to 0.84% in Central regions and 0.64% in Western regions. The difference of 0.67 percentage points between Eastern and Western regions represents substantial regional disparity, supporting the rationale for heterogeneity analysis. Research on government effects on health system efficiency during China’s new health reform has similarly emphasized regional stratification (38).

**Table 3:** Descriptive statistics by region.

Region	Obs	LE (mean)	LE (S.D.)	SI (mean)	SI (S.D.)	Provinces
Eastern	165	78.65	2.14	1.31	0.52	11
Central	120	76.92	1.87	0.84	0.28	8
Western	165	74.18	2.56	0.64	0.24	11
Full sample	450	76.58	2.79	0.94	0.45	30

The panel structure (*N* = 30, *T* = 15) warrants methodological discussion. While the time dimension is relatively short, the coefficient of variation in SI (0.48) indicates sufficient variation for threshold identification. The fixed effects specification controls for time-invariant provincial heterogeneity, and year effects absorb common temporal shocks. The relatively short panel limits the ability to fully separate long-run equilibrium relationships from transitional dynamics, and the results should be interpreted as capturing medium-term associations rather than definitive long-run causal effects.

### 4.4 Econometric approach

This study employs Hansen's (39) panel threshold regression model to examine the nonlinear relationship between sports industry development and public health. The model allows regression coefficients to differ across regimes defined by estimated threshold values, enabling detection of structural breaks in the relationship of interest (40). The basic single-threshold panel model takes the following form:


$$\begin{array}{l} LE_{\mathrm{it}}=\mu_{i}+\lambda_{t}+\beta_{1}SI_{\mathrm{it}}\cdot I(SI_{\mathrm{it}}\leq \gamma )\\ +\beta_{2}SI_{\mathrm{it}}\cdot I(SI_{\mathrm{it}}>\gamma )+\theta X_{\mathrm{it}}+\varepsilon_{\mathrm{it}} \end{array}$$ (1)

Where $LE_{\mathrm{it}}$ represents life expectancy at birth for province i in year t ; $SI_{\mathrm{it}}$ denotes sports industry development measured as percentage of GDP; γ is the threshold value to be estimated; I(⋅) is an indicator function that equals 1 when the condition is satisfied and 0 otherwise; $X_{\mathrm{it}}$ is a vector of control variables; $\mu_{i}$ captures province fixed effects absorbing time-invariant unobserved heterogeneity; $\lambda_{t}$ represents year fixed effects controlling for common temporal shocks; and $\varepsilon_{\mathrm{it}}$ is the idiosyncratic error term. The coefficients $\beta_{1}$ and $\beta_{2}$ represent the effects of sports industry development on public health in the low regime ( $SI_{\mathrm{it}}\leq \gamma$ ) and high regime ( $SI_{\mathrm{it}}>\gamma$ ), respectively. A statistically significant difference between $\beta_{1}$ and $\beta_{2}$ indicates the presence of threshold effects.

The threshold value γ is estimated by minimizing the concentrated sum of squared residuals across all candidate threshold values within the 5th to 95th percentile range of the threshold variable distribution:

(2) $$\hat{\gamma }=\underset{\gamma }{\text{argmin}}\;\;S_{1}(\gamma )$$

Where S(γ) denotes the sum of squared residuals at threshold value γ . The statistical significance of threshold effects is assessed using bootstrap methods with 1,000 iterations. The likelihood ratio test statistic is employed to test the null hypothesis of no threshold effects:

(3) $$\mathrm{LR}(\gamma )=\frac{S_{1}(\gamma )-S_{1}(\hat{\gamma })}{{\hat{\sigma }}^{2}}$$

The 95% confidence interval of the threshold parameter is constructed by finding values that produce a likelihood ratio statistic below the critical value of 7.35. For multiple thresholds, a sequential testing approach is adopted: the single threshold model is tested against the linear model, and if significant, the double threshold model is tested against the single threshold model.

Endogeneity may arise from reverse causality or omitted variables. Regions with healthier populations may attract greater investment in sports infrastructure, and unobserved factors such as regional governance quality may jointly influence both sports industry development and health outcomes. To address these concerns, instrumental variable estimation and system GMM estimation are employed as robustness checks, with detailed results reported in Section 5.4.

## 5 Empirical results

### 5.1 Unit root tests

Before the threshold values are estimated, panel unit root tests are used to check the stationarity of all variables. The results are displayed in Table 4, which includes three different tests: the Levin-Lin-Chu (LLC) test assuming common unit root processes, the Im-Pesaran-Shin (IPS) test allowing for heterogeneous processes, and the Fisher-ADF test combining *p*-values from individual unit root tests. The results reject the unit root null hypothesis for most variables at conventional significance levels in at least two of three tests. Given the relatively short time dimension (*T* = 15) and the panel threshold regression’s robustness to non-stationarity under certain conditions, the analysis proceeds with level variables while including both province and year fixed effects (41).

**Table 4:** Panel unit root test results.

Variable	LLC test	IPS test	Fisher-ADF test
LE	−4.562^***^	−2.876^***^	98.45^***^
SI	−3.845^***^	−2.134^**^	86.32^***^
lnPGDP	−2.156^**^	−1.654^*^	72.18^**^
ME	−3.478^***^	−2.345^***^	89.67^***^
EDU	−4.125^***^	−2.567^***^	94.23^***^
URB	−2.876^***^	−1.892^**^	78.56^**^
PM25	−5.234^***^	−3.156^***^	112.45^***^
AGE	−3.567***	−2.234^**^	82.34^***^
MR	−4.012***	−2.678^***^	91.78^***^

^***^, ^**^, ^*^ denote significance at 1, 5, and 10% levels, respectively.

### 5.2 National-level threshold analysis

Table 5 shows the threshold effects tests conducted on the national sample. The single threshold test has an *F*-statistic of 52.86 and a *p*-value of 0.027 from the bootstrapped test, thus rejecting the null hypothesis of no threshold at the 5% significance level. The value of the threshold is *γ* = 0.96% of GDP, and the 95% confidence interval is [0.89, 1.04]. The double threshold test has an *F*-statistic of 9.38 and an unadjusted *p*-value of 0.856, thus failing to reject the null hypothesis of a single threshold. These results indicate that the relationship between sports industry development and public health exhibits a single threshold at the national level, with an econometric regime shift occurring at approximately 0.96% of GDP (42, 43). This threshold should be interpreted as a statistical breakpoint in the estimated relationship rather than a precise policy target.

**Table 5:** Threshold effect test results (national level).

Test	*F*-statistic	*p*-value	Threshold (*γ*)	95% CI
Single threshold	52.86^**^	0.027	0.96	[0.89, 1.04]
Double threshold	9.38	0.856	—	—

Bootstrap replications = 1,000. ^**^ denotes significance at 5% level.

The results of the threshold regression are shown in Table 6. Based on Equation 1, in the low regime (SI ≤ 0.96%), the positive coefficient on sports industry development has a magnitude of 0.312 (*p* < 0.01), which shows that an increase of one percentage point in the share of the sports industry results in enhancing life expectancy by about 0.31 years (about 3.7 months). In the high regime (SI > 0.96%), the coefficient increases to 0.369 (*p* < 0.01), representing an 18.3% enhancement in the promotion effect. This supports the conclusion of Hypothesis 2, and it also holds that the health benefits of the development of the sports industry are strengthened once the threshold level is passed (44). The strength of such effects is similar to that found in other studies of the relationship between economic factors and health that were conducted in developing nations and had coefficients of roughly similar GDP share variables between 0.2 and 0.5. It would appear that the enhanced impact found in the high-regime equation is due to the effects of network externalities and accumulated health awareness.

**Table 6:** Threshold regression results (national level).

Variable	Coefficient	Std. error
SI (SI ≤ 0.96%)	0.312^***^	0.086
SI (SI > 0.96%)	0.369^***^	0.072
lnPGDP	2.345^***^	0.312
ME	0.0004^***^	0.0001
EDU	0.456^***^	0.089
URB	0.034^**^	0.015
PM25	−0.028^***^	0.008
AGE	−0.156^***^	0.042
MR	0.234^***^	0.056
Province FE	Yes	
Year FE	Yes	
R-squared	0.892	
Observations	450	

^***^, ^**^, ^*^ denote significance at 1, 5, and 10% levels, respectively.

**Figure 3:**
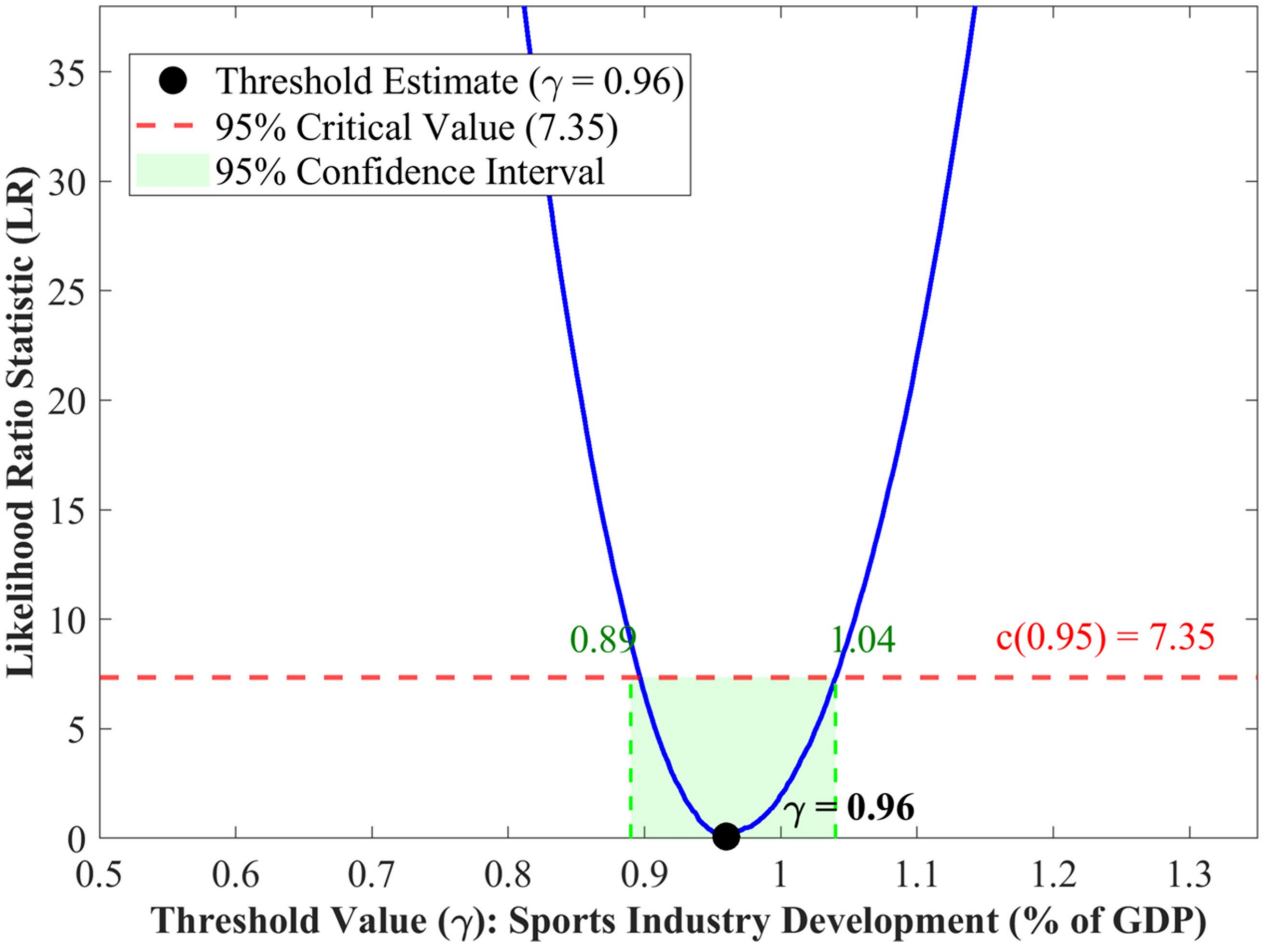
LR test for threshold value—national level.

To visualize the threshold identification process, Figure 3 presents the likelihood ratio test statistic as a function of potential threshold values for the national sample. As shown in Figure 3, the LR curve reaches the minimum at γ = 0.96, indicating the optimal threshold estimate obtained through Equation (2). The 95% confidence interval is constructed using Equation 3 by selecting the thresholds for which the LR statistic is below the critical value of 7.35 (indicated by the horizontal dashed line), resulting in the range [0.89, 1.04] (shaded region). This narrow range indicates a sharp estimate of the value of the threshold parameter. The LR curve follows a U-shape, indicating sharp slopes in the margins of the minimum, pointing to strong statistical identification of the threshold effect. The asymmetry of the LR curve, where the left side slopes a little more sharply than the right side, implies that the cost of departure from the threshold is more for the former than the latter.

The magnitude of estimated effects warrants cautious interpretation. The coefficients are larger than those typically reported in cross-country health economics studies, which may partly reflect aggregation at the provincial level. Life expectancy captures cumulative health improvements influenced by multiple correlated factors beyond sports industry development alone. Additionally, the relatively short panel (*T* = 15) limits the ability to fully disentangle long-run causal effects from transitional dynamics. The threshold effects should be understood as identifying regime shifts in the statistical relationship rather than precise causal impacts.

The control variables show relevant signs consistent with existing literature. Economic development (lnPGDP) has a significant positive effect on life expectancy (coefficient: 2.345, *p* < 0.01), consistent with the gradient of health to wealth reported in cross-country studies. The large coefficient could be attributed to China’s fast economic development in which GDP expansion has occurred in tandem with marked improvements in nutrition, healthcare access, and living standards. Medical expenditure (ME) and education level (EDU) have a positive influence on health outcomes, emphasizing the importance of healthcare access and human capital. The Urbanization (URB) has a small positive influence (0.034, *p* < 0.05), while air pollution, as indicated by PM2.5, has a statistically significant negative influence (−0.028, *p* < 0.01), consistent with epidemiological evidence regarding health outcomes and air pollution. Population aging (AGE) is negatively related to life expectancy (−0.156, *p* < 0.01), indicating a compositional effect due to the aging population. Medical resource availability (MR) has a strong positive association (0.234, *p* < 0.01).

### 5.3 Regional heterogeneity analysis

In order to evaluate the heterogeneity of threshold effects at the regional level, separate tests are employed for Eastern, Central, and Western China. Results of the threshold effects tests by region are shown in Table 7. Regionally, Eastern China does not have a significant threshold effect (*F* = 11.28, *p* = 0.426), indicating the presence of a linear relationship between sports industry development and public health in the most developed region. This could be indicative of the matured status of the sports industry in Eastern China, where the best infrastructure and maximum participation have been attained. On the contrary, the single threshold effects are significant for the Central as well as the Western regions. The Central region threshold is estimated at *γ* = 1.06% (95% CI: [0.97, 1.14]), while the Western region threshold is *γ* = 0.79% (95% CI: [0.72, 0.87]) (45).

**Table 7:** Threshold effect test results by region.

Region	Obs	*F*-statistic	*p*-value	Threshold (*γ*)	95% CI
Eastern	165	11.28	0.426	—	—
Central	120	36.42^**^	0.038	1.06	[0.97, 1.14]
Western	165	40.75^**^	0.024	0.79	[0.72, 0.87]

Bootstrap replications = 1,000. ^**^ denotes significance at 5% level. Eastern region shows no significant threshold.

The ordering of threshold values—Western (0.79) < National (0.96) < Central (1.06)—warrants cautious interpretation given the relatively small regional subsamples. The lower Western threshold may reflect earlier development stages where basic infrastructure investments yield initial health gains, though this interpretation remains tentative and requires validation with larger samples and longer time series. The higher Central China threshold, despite the relatively lower stages of development, could be indicative of the transitional dynamics specific to that region, where the provinces already have adequate health infrastructure support to address fundamental health requirements but require more advanced sports industry development to maximize the effects of network externalities and economy-of-scale effects that snowball health outcomes to achieve accelerated results. This pattern is consistent with an S-curve developmental model where the midpoint regions experience transient bottlenecks before recording breakthrough results. These interpretations remain tentative given the limited regional subsamples, and further research with longer time series would help clarify the institutional mechanisms underlying these regional patterns.

Table 8 shows the results of the threshold regression model by region. For Eastern China, without any threshold existing in the region, a standard fixed effects model indicates a coefficient of 0.245 (*p* < 0.01) for sports industry development. This coefficient is lower than the post-threshold values observed in Central (0.324) and Western (0.225) regions, suggesting diminishing marginal health returns in provinces where sports infrastructure has already reached relatively high levels of development. For Central China, the promotion effect enhances by 13.3% from 0.286 in the low state to 0.324 in the high state for a 1.06% threshold value. The Western region demonstrates the largest boost in the value of the coefficients from 0.178 to 0.225, corresponding to an increase of 26.4%. This clearly supports the “timely assistance” hypothesis in terms of health returns in the sense that sports investment maximizes health returns in less developed regions.

**Table 8:** Threshold regression results by region.

Variable	Eastern	Central	Western
SI (low regime)	0.245^***^ (0.073)	0.286^***^ (0.092)	0.178^***^ (0.064)
SI (high regime)	—	0.324^***^ (0.085)	0.225^***^ (0.071)
Effect enhancement	Linear	+13.3%	+26.4%
Threshold (*γ*)	Not significant	1.06%	0.79%
Control variables	Yes	Yes	Yes
Province FE	Yes	Yes	Yes
Year FE	Yes	Yes	Yes
R-squared	0.876	0.902	0.885
Observations	165	120	165

^***^ denotes significance at 1% level. Standard errors in parentheses. Control variables include lnPGDP, ME, EDU, URB, PM25, AGE, and MR.

**Figure 4:**
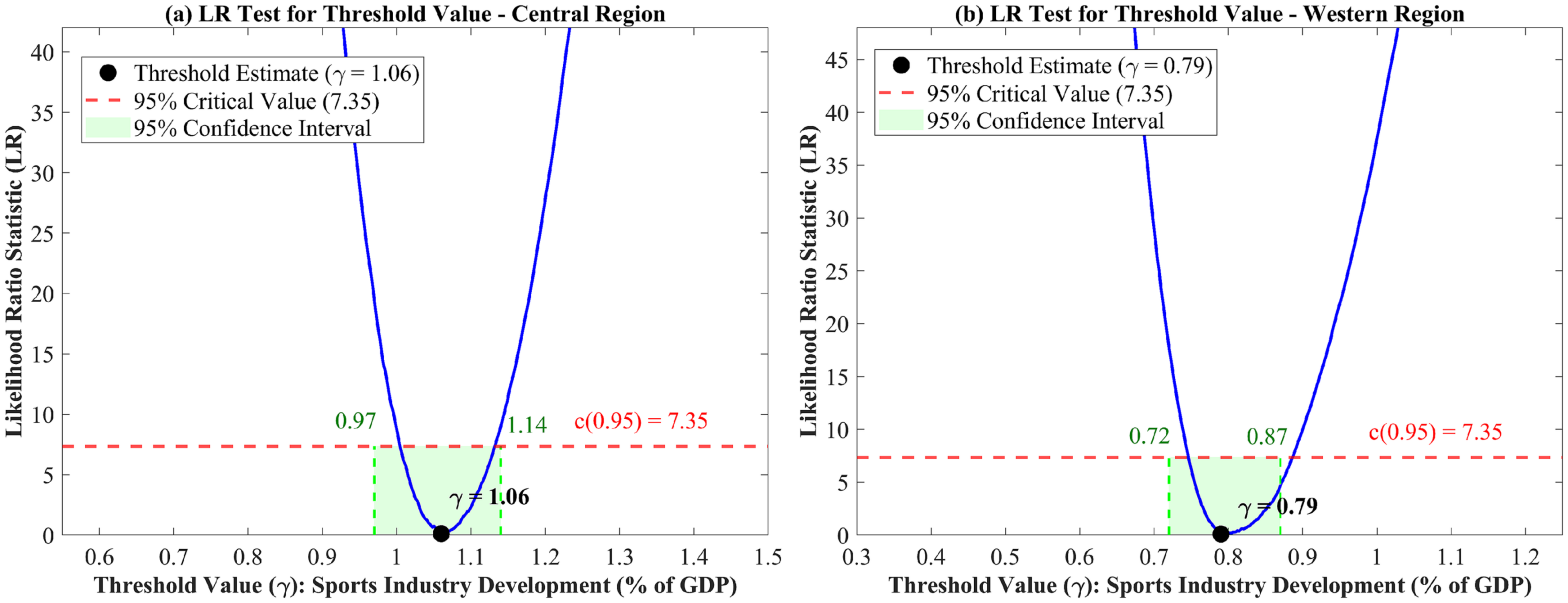
LR test for threshold value by region: **(a)** Central region; **(b)** Western region.

Figure 4 displays the LR test statistics for Central and Western regions, illustrating the threshold identification process in each case. In Figure 4a, the analysis on the Central region is shown, with the smallest LR being at *γ* = 1.06. Here, the confidence interval is [0.97, 1.14], which is larger than the previous one due to the smaller scale (*N* = 120). Figure 4b contains the data on the Western region. Here, the threshold is at *γ* = 0.79, with the confidence interval being [0.72, 0.87]. The LR function in the Western region is steeper on the left than on the right. This suggests that the relationship on the left is much weaker than on the right. This pattern indicates that once sports industry development in Western regions surpasses 0.79%, health promotion effects increase substantially.

### 5.4 Robustness checks

Several robustness checks are conducted to validate the main findings and address potential endogeneity concerns. Endogeneity may arise from reverse causality, as regions with healthier populations may attract greater investment in sports infrastructure. Additionally, unobserved factors such as regional governance quality or health culture may jointly influence both sports industry development and health outcomes. To address these issues, five alternative specifications are examined, as shown in Table 9. The analysis includes alternative dependent variable (healthy life expectancy), alternative independent variable (per capita sports consumption), sample restriction (excluding four municipalities), instrumental variable estimation, and temporal sensitivity test (excluding 2020).

**Table 9:** Robustness check results.

Specification	Threshold	*β*₁ (S.E.)	*β*₂ (S.E.)	Enhancement	Controls	FE
Baseline (LE)	0.96^**^	0.312^***^ (0.086)	0.369^***^ (0.072)	+18.3%	Yes	Yes
Alternative DV (HLE)	0.94^**^	0.285^***^ (0.089)	0.341^***^ (0.076)	+19.6%	Yes	Yes
Alternative IV (consumption)	0.92^**^	0.298^***^ (0.091)	0.346^***^ (0.078)	+16.1%	Yes	Yes
Exclude municipalities	0.98^**^	0.305^***^ (0.098)	0.362^***^ (0.084)	+18.7%	Yes	Yes
IV estimation (2SLS)	0.95^**^	0.287^***^ (0.105)	0.342^***^ (0.093)	+19.2%	Yes	Yes
Exclude 2020 (COVID)	0.97^**^	0.318^***^ (0.088)	0.375^***^ (0.075)	+17.9%	Yes	Yes

^***^, ^**^ denote significance at 1 and 5% levels, respectively. Threshold significance tested by bootstrap with 1,000 replications. Standard errors in parentheses. Controls include lnPGDP, ME, EDU, URB, PM25, AGE, and MR. FE = province and year fixed effects. HLE = healthy life expectancy.

All alternative specifications show significant threshold effects with similar coefficient patterns, supporting the robustness of the main findings. The IV estimates are approximately 8% smaller than the baseline results (0.287 versus 0.312 for *β*₁), consistent with the expectation that OLS estimates may be upwardly biased due to reverse causality. The Hansen J-test does not reject the null hypothesis of instrument validity (*p* = 0.342), and the Arellano-Bond AR(2) test confirms no second-order serial correlation (*p* = 0.278). Excluding the COVID-19 year produces nearly identical results, suggesting that pandemic-related disruptions do not substantially affect the threshold estimates.

To further assess the validity of the instrumental variable estimation, Table 10 reports the first-stage regression results. The one-period lagged sports industry development and provincial sports fiscal expenditure serve as instruments. The coefficient on lagged SI is 0.823 (*p* < 0.01), indicating strong persistence in sports industry development. The sports fiscal expenditure coefficient is positive and significant at the 5% level (0.019), though smaller in magnitude. The first-stage *F*-statistic for excluded instruments is 16.45. While this exceeds the conventional threshold of 10, it falls below the Stock-Yogo critical value of 19.93 for 10% maximal IV size, suggesting that weak instrument bias cannot be entirely ruled out. The Kleibergen-Paap rk LM statistic rejects the null hypothesis of underidentification (*p* < 0.01). Given these diagnostics, the IV results should be interpreted with some caution, though the consistency between IV and baseline estimates suggests that endogeneity does not fundamentally alter the main findings.

**Table 10:** First-stage results for instrumental variable estimation.

Variable	Coefficient	Std. error	*t*-statistic
SI (*t*-1)	0.823^***^	0.054	15.24
Sports fiscal expenditure	0.019^**^	0.008	2.38
lnPGDP	0.156^***^	0.048	3.25
ME	0.00002	0.00002	1.00
EDU	0.024	0.018	1.33
URB	0.003^**^	0.001	3.00
PM25	−0.001	0.001	−1.00
AGE	−0.008	0.007	−1.14
MR	0.005	0.009	0.56
Province FE	Yes		
Year FE	Yes		
Observations	420		
R-squared	0.876		
*F*-statistic (excluded instruments)	16.45		
Kleibergen-Paap rk LM statistic	21.87^***^		
Stock-Yogo weak ID critical value (10%)	19.93		

^***^, ^**^ denote significance at 1 and 5% levels, respectively. Dependent variable is SI. The *F*-statistic is below the Stock-Yogo critical value, suggesting potential weak instrument concerns.

To further check the validity of the estimated effects, these are contrasted with those in previous studies in the literature. Previous studies on growth and health relationships in China estimated that a one percentage point increase in GDP growth is linked to 0.2–0.4-year gains in life expectancy, depending on models and time horizons investigated (17, 18). The sports sector-specific effects estimated in this study (0.31–0.37 years per percentage point of GDP) are seen to be consistent with previous studies, and further, the 18–19% increase at the threshold level appears to be consistent with previous studies on health relationships and public expenditures (5).

## 6 Conclusion

This study examines the relationship between sports industry development and public health across 30 Chinese provinces from 2010 to 2024 using Hansen’s panel threshold regression model. Three main findings emerge from the analysis.

The sports industry development has a significant positive effect on public health, supporting Hypothesis 1. At the national level, a single threshold is identified at 0.96% of GDP, with the health-promoting effect increasing by 18.3% once this threshold is crossed, supporting Hypothesis 2. Regional heterogeneity is substantial, supporting Hypothesis 3: Eastern China exhibits a linear relationship without significant thresholds; Central China shows a higher threshold at 1.06% with 13.3% effect enhancement; and Western China demonstrates the lowest threshold at 0.79% with the strongest post-threshold enhancement of 26.4%. These results remain robust across alternative specifications and instrumental variable estimation.

The findings suggest differentiated regional strategies. Western provinces stand to gain most from targeted sports industry investment given lower thresholds and larger post-threshold effects. Central provinces require continued development to surpass their higher threshold, while Eastern provinces may benefit more from improvements in service quality and community accessibility than from aggregate expansion.

Several limitations should be noted. Provincial-level data may mask within-province variations; city-level analysis could provide more granular insights. The threshold model captures nonlinearities but does not empirically verify the hypothesized mediating pathways. The instrumental variables face inherent limitations, and while estimates are consistent across specifications, quasi-experimental designs would strengthen causal identification. International comparative analysis could assess whether these threshold patterns generalize beyond the Chinese context.

## Data Availability

Publicly available datasets were analyzed in this study. This data can be found at: The datasets analyzed in this study are publicly available from the following sources: National Bureau of Statistics of China: https://www.stats.gov.cn/, China Statistical Yearbook: https://www.stats.gov.cn/sj/ndsj/, China Health Statistical Yearbook: http://www.nhc.gov.cn/ and General Administration of Sport of China: https://www.sport.gov.cn/.
